# Genomics of Postprandial Lipidomics in the Genetics of Lipid-Lowering Drugs and Diet Network Study

**DOI:** 10.3390/nu13114000

**Published:** 2021-11-10

**Authors:** Marguerite R. Irvin, May E. Montasser, Tobias Kind, Sili Fan, Dinesh K. Barupal, Amit Patki, Rikki M. Tanner, Nicole D. Armstrong, Kathleen A. Ryan, Steven A. Claas, Jeffrey R. O’Connell, Hemant K. Tiwari, Donna K. Arnett

**Affiliations:** 1 Department of Epidemiology, University of Alabama at Birmingham, Birmingham, AL 35294, USA; rmdeitz@uab.edu (R.M.T.); nmda@uab.edu (N.D.A.); 2 Department of Medicine, Division of Endocrinology, Diabetes, and Nutrition, University of Maryland School of Medicine, Baltimore, MD 21201, USA; mmontass@som.umaryland.edu (M.E.M.); Kathleen.Ryan@som.umaryland.edu (K.A.R.); joconnel@som.umaryland.edu (J.R.O.); 3 Program for Personalized and Genomic Medicine, University of Maryland School of Medicine, Baltimore, MD 21201, USA; 4 NIH West Coast Metabolomics Center, UC Davis Genome Center, University of California, Davis, CA 95616, USA; tkind@ucdavis.edu (T.K.); fansili2013@gmail.com (S.F.); 5 Department of Environmental Medicine and Public Health, Icahn School of Medicine at Mount Sinai, New York, NY 10029, USA; dinesh.kumar@mssm.edu; 6 Department of Biostatistics, University of Alabama at Birmingham, Birmingham, AL 35294, USA; apatki@uab.edu (A.P.); htiwari@uab.edu (H.K.T.); 7 College of Public Health, University of Kentucky, Lexington, KY 40536, USA; steven.claas@uky.edu (S.A.C.); donna.arnett@uky.edu (D.K.A.)

**Keywords:** lipidomics, genomics, *FADS1*, *FADS2*, postprandial

## Abstract

Postprandial lipemia (PPL) is an important risk factor for cardiovascular disease. Inter-individual variation in the dietary response to a meal is known to be influenced by genetic factors, yet genes that dictate variation in postprandial lipids are not completely characterized. Genetic studies of the plasma lipidome can help to better understand postprandial metabolism by isolating lipid molecular species which are more closely related to the genome. We measured the plasma lipidome at fasting and 6 h after a standardized high-fat meal in 668 participants from the Genetics of Lipid-Lowering Drugs and Diet Network study (GOLDN) using ultra-performance liquid chromatography coupled to (quadrupole) time-of-flight mass spectrometry. A total of 413 unique lipids were identified. Heritable and responsive lipid species were examined for association with single-nucleotide polymorphisms (SNPs) genotyped on the Affymetrix 6.0 array. The most statistically significant SNP findings were replicated in the Amish Heredity and Phenotype Intervention (HAPI) Heart Study. We further followed up findings from GOLDN with a regional analysis of cytosine-phosphate-guanine (CpGs) sites measured on the Illumina HumanMethylation450 array. A total of 132 lipids were both responsive to the meal challenge and heritable in the GOLDN study. After correction for multiple testing of 132 lipids (α = 5 × 10^−8^/132 = 4 × 10^−10^), no SNP was statistically significantly associated with any lipid response. Four SNPs in the region of a known lipid locus (fatty acid desaturase 1 and 2/*FADS1* and *FADS2*) on chromosome 11 had *p* < 8.0 × 10^−7^ for arachidonic acid FA(20:4). Those SNPs replicated in HAPI Heart with *p* < 3.3 × 10^−3^. CpGs around the *FADS1/2* region were associated with arachidonic acid and the relationship of one SNP was partially mediated by a CpG (*p* = 0.005). Both SNPs and CpGs from the fatty acid desaturase region on chromosome 11 contribute jointly and independently to the diet response to a high-fat meal.

## 1. Introduction

Most individuals experience continuous fluctuations in postprandial lipemia (PPL) throughout the day [1]. Results from basic and clinical studies suggest that postprandial lipid levels are associated with prothrombotic and proinflammatory processes [2] related to cardiovascular disease (CVD) [3,4,5]. Remnant postprandial particles are partially catabolized chylomicrons and very low-density lipoproteins (VLDLs) that are reduced in size, partially depleted of triglycerides (TGs), and enriched with cholesteryl esters (CEs) [6]. Remnant particles that remain in the circulation for an extended time are independently related to coronary artery disease progression [7,8,9]. Overall, quantifying the CVD risk associated with PPL has been challenged by the absence of these measures in large observational studies and the need for standardization of the meal prior to the lipid measures. Small, controlled studies of response to a high-fat meal have shown striking inter-individual variation and report that a more pronounced PPL response followed by delayed lipid clearance predicts the presence of CVD [10,11,12,13,14]. Overall, studies show lipids peak an average 4–6 h following a meal [7,10,15]. Lipid levels measured 6 h after an intervention are discriminatory for the presence of CVD [10,16]. Additionally, research has shown PPL lipid levels are heritable; thus, genes may help better understand PPL processes [17,18]. A genome-wide association study (GWAS) of PPL has shown a locus near the apolipoprotein gene cluster (*APOA1/C3/A4/A5*) on chromosome 11 was associated with the postprandial TG response [19]. An epigenome-wide association study (EWAS) of postprandial TG has similarly highlighted known lipid-related genes [20]. Still, complex biology underlies these responses, and more granular lipid phenotypes may continue to help unravel proinflammatory mechanisms related to diet [21].

Lipidomics in epidemiological settings characterizes the composition of lipid molecular species in a population [22]. High throughput lipidomics research has been largely driven by advances in mass spectrometry (MS) and chromatographic technologies. Many studies have already demonstrated the utility of lipidomics for biomarker discovery related to CVD [23,24]. Continued research is needed to determine if lipidomic species could eventually complement conventional lipid measures currently used in the clinic [25,26,27].

The constituents of the lipidome are proving to be “closer to the genome” than traditional lipid measurements [28,29]. For example, a targeted lipidomics study by Hicks et al. reported circulating concentrations of several key molecules involved in sphingolipid metabolism were strongly associated with common genetic variants (*p*-values = 10^−15^ to 10^−66^) [30]. GWAS of fasting lipidomic traits in the Cooperative Health Research in the Region Augsburg (KORA) study highlighted single nucleotide polymorphisms (SNPs) located in or near genes central to lipid metabolism with *p*-values ranging 3 × 10^−24^ to 6.5 × 10^−179^ [31]. However, few studies have examined genomic factors associated with the postprandial lipidome. In the current study, we conducted lipidomic analysis at two time points (before and 6 h after a standardized high-fat meal) in over 600 participants from the Genetics of Lipid-Lowering Drugs and Diet Network (GOLDN) study. We identified diet-responsive and heritable lipids and further conducted GWAS of those lipid candidates. We replicated the genetic results in the Heredity and Phenotype Intervention (HAPI) Heart Study [32]. Finally, we examined methylation markers and methylation quantitative trait loci (meQTLs) around the GWAS-identified candidates of interest.

## 2. Materials and Methods

### 2.1. Study Population

GOLDN participants were identified through 3-generation families previously screened in the NHLBI Family Heart Study Minnesota or Utah centers [33]. As part of the NHLBI Programs in Gene by Environment Interaction (PROGENI), participants took part in diet and/or drug interventions (high-fat meal and/or fenofibrate for 3 weeks) and provided consent for genetic studies. GOLDN participants who were using lipid-lowering drugs were required to discontinue them for 4 weeks prior to the study. The GOLDN sample consisted of 1048 Caucasian individuals belonging to 184 pedigrees (average family size 6.2). A total 668 of those participants had all available ‘omics’ data to support the current study. The study was approved by the Institutional Review Board at the University of Alabama at Birmingham (UAB). The current study focuses on the diet intervention arm of the study only.

### 2.2. High-Fat Meal and Measurement of Postprandial Lipemia

Participants fasted for ≥12 h and abstained from alcohol intake for ≥24 h. The PPL intervention followed the protocol of Patsch et al. [10]. The whipping cream (83% fat) meal had 700 calories/m^2^ body surface area; 3% of calories were derived from protein and 14% from carbohydrates. Blood samples were drawn immediately before (fasting) and at 6 h after consuming the high-fat meal. During the 6-h study period, participants consumed only water and abstained from physical activity.

### 2.3. Genomics

GOLDN participants were genotyped using the Affymetrix Genome-Wide Human SNP Array 6.0 (Thermo Fisher Scientific, Waltham, MA, USA). DNA extraction and purification were done using commercial Puregene reagents (Gentra System, Inc, Minneapolis, MN, USA) as described in Irvin et al. [34]. We typed 906,600 SNPs and called them using the Birdseed calling algorithm. Comprehensive quality control (QC) procedures have been described for GOLDN [19]. Due to the volume of GWAS models for lipidomic outcomes, we used the QC’d version of the genotyped data to minimize computing time and resources. Methylation assays were performed using Illumina’s Infinium Human Methylation 450 Beadchip [35] (Illumina, Inc, San Diego, CA, USA) in GOLDN on DNA extracted from T-cells derived from buffy coat samples. The GOLDN methylation assays and QC have been described [36].

### 2.4. Lipidomics

GOLDN lipidomics data were collected using ultra-performance liquid chromatography coupled to (quadrupole) time-of-flight mass spectrometry (UPLC–QTOFMS) at the West Coast Metabolomics Center (WCMC) at the University of California Davis. The protocol for this measurement has been described in detail [37,38]. In summary, the process was divided into three steps: lipid extraction and separation, data acquisition, and lipid identification. Methyl *tert*-butyl ether, methanol, and water were used for plasma lipid extraction. Blanks and pooled human plasma were used for QC samples (BioreclamationIVT, Westbury, NY, USA). The separated non-polar phase was injected into a Waters Acquity UPLC CSH C18 (100 mm length × 2.1 mm id; 1.7 μm particle size, Waters Corp, Milford, MA, USA) with an additional Waters Acquity VanGuard CSH C18 pre-column (5 mm × 2.1 mm id; 1.7 μm particle size) maintained at 65 °C coupled to an Agilent 1290 Infinity UHPLC (Agilent Technologies, Inc, Santa Clara, CA, USA) for electrospray ionization (ESI) positive and negative modes. Mobile phase modifiers included ammonium formate and formic acid for positive mode and ammonium acetate for negative mode (MilliporeSigma, Burlington, MA, USA). For both positive and negative modes, the same mobile phase composition of (A) 60:40 *v/v* acetonitrile:water (liquid chromatography-mass spectrometry [MS] grade) and (B) 90:10 *v/v* isopropanol:acetonitrile was used. An Agilent 6550 QTOF with a jet stream electrospray source was employed for acquiring full scan data in the mass range m/z 65–1700 in positive and negative modes with a scan rate of 2 spectra/s. Instrument parameters were as follows for the ESI (+) mode—gas temperature 325  °C, gas flow 8 L/min, nebulizer 35 psig, sheath gas temperature 350  °C, sheath gas flow 11, capillary voltage 3500  V, nozzle voltage 1000  V, fragmentor voltage 120  V, and skimmer 65  V. In negative ion mode, gas temperature was 200 °C, gas flow 14  L/min, fragmentor 175  V, with the other parameters identical to positive ion mode. Data were collected in centroid mode at a rate of 2 scans/s. Injection volumes were 1.7  μL and 5  μL for the positive and negative modes, respectively. The gradient started at 15% B, ramped to 30% at 2 min, 48% at 2.5 min, 82% at 11 min, 99% at 11.5 min, and kept at 99% B until 12 min before ramping down to 15% B at 12.1  min, which was kept isocratic until 15  min to equilibrate the column. Run time was 15 min and the flow rate was 0.6 mL/min. For every ten samples, one QC sample was analyzed. Data were acquired in nine batches. We collected MS1 data for all samples, and we collected MS/MS data for a set of pooled samples. Data were processed with the Agilent Quant 7.0 software. Lipid levels were reported as chromatographic peak heights and the data were normalized using the SERRF method [39]. After normalization, the relative standard deviation (SD) of QC samples was 4.7% and 3.4% for negative and positive modes, respectively. Lipid identification was performed by converting the acquired MS/MS spectra to the mascot generic format (MGF) and then conducting a library search using the in-silico MS/MS library LipidBlast [40]. In total, 413 lipids were characterized including CEs, lysophosphatidylcholines (LPCs), phosphatidylcholines (PCs), phosphatidylethanolamines (PEs), lysophosphatidylethanolamines (LPEs), sphingomyelins (SMs), phosphatidlyserines, phosphatidylinositols (PIs), ceramides, and TGs [41].

### 2.5. Replication Population

The samples used in this study were from participants of the HAPI Heart Study [32]. Briefly, HAPI Heart was initiated in 2002 to identify the genetic and environmental determinants of responses (blood pressure, TG excursion and platelet aggregation) to four short-term interventions which included a high-fat challenge. Baseline blood drawn from 650 participants was used for the lipidomic profiling in this study. Since both GOLDN and HAPI Heart were part of the NHLBI PROGENI program, the diet intervention was harmonized between the studies. The study protocol was approved by the Institutional Review Board at the University of Maryland. Informed consent was obtained from each of the study participants. Genome-wide genotyping was performed with the Affymetrix GeneChip Mapping 500 K Assay at the University of Maryland Biopolymer Core Facility. QC has been described, resulting in the inclusion of 307,238 genotyped variants for imputation [42]. The genotype data were uploaded to the Michigan Imputation Server [43] where the pre-phasing was performed using Eagle v2.4 [44], and then imputation to the TOPMed Freeze 5b reference panel was performed using Minimac4 [45]. Following imputation, variants with imputation quality/INFO  < 0.9, minor allele frequency < 0.0001, or deviation from Hardy-Weinberg equilibrium at *p* < 1.0 × 10^−9^ were excluded. Finally, the same lipidomics assays were measured on 639 HAPI Heart participants at the WCMC [41].

## 3. Statistical Methods

Descriptive Statistics: Participant characteristics in GOLDN and HAPI Heart were reported as mean (±SD) or counts and percent as appropriate.

Responsive and Heritable Lipids: Using a linear mixed model, we tested whether the intercept for each lipidomic trait change (6 h-fasting value) measured in GOLDN was statistically different from zero after adjustment for the fasting value, age, sex, and family ID (modeled as a random effect). Heritability (h^2^) estimates of lipidomic traits were estimated using a variation of the linear mixed model with GWAS data as previously described [46,47]. Lipids that changed (*p* < 0.05) in response to the PPL intervention and had h^2^ ≥ 20% were included in genetic analysis.

Genetic Analysis: PPL response was defined as the change or delta in the lipidomic trait value from the fasting value. Each delta was inverse rank-based transformed. We used linear regression implemented in PLINK (v1.9) for detecting the association between SNP genotypes and lipidomic trait outcomes. Covariates included age, gender, study site, and fasting lipidomic concentration. As population substructure within GOLDN is minimized by the design (Caucasian families from MN and UT), we did not adjust for ancestry. Inflation corrected *p*-values are presented in Manhattan and QQ plots in Supplemental Files. In sensitivity models for the top hits, the family structure was modeled as a random effect by using the LMEKIN R package [48]. We followed up the top GWAS findings (*p* < 1.0 × 10^−7^) with a regional analysis of cytosine-phosphate-guanine sites (CpGs, annotated to the GWAS SNP gene location or a ±20 kb region around the SNP, if intergenic). For epigenetic association, we took a two-step approach. We first obtained the residuals from a model regressing four methylation principal components (PCs) onto the CpG. We then regressed the CpG residual onto the PPL response trait, adjusting for the same covariates as in the GWAS. For CpGs with association *p*-value with the lipid trait of interest <0.01, we examined meQTLs. For meQTL analysis, we regressed the nearest GWAS SNP onto the CpG, adjusting for age, sex, and methylation PCs. For top findings from the CpG analysis qualifying as a meQTL (*p* < 10^−9^ based on prior work in GOLDN) [49], mediation analysis was used to determine if the association between the GWAS SNP and lipid was mediated by the CpG. Mediation analysis was performed in SAS, version 9.4 (SAS Institute Inc. Cary, NC, USA). The models were adjusted for age, sex, center, and baseline/fasting lipid. Replication analysis in HAPI Heart for 8 of the top SNPs was performed similarly to GOLDN where each SNP was regressed on the inverse normally transformed lipid change adjusting for age, sex, baseline level, and genetic relationship matrix using Mixed Model Analysis for Pedigree and population (MMAP) (https://github.com/MMAP DOI:10.5281/zenodo.5033491, accessed on 27 July 2021).

## 4. Results

**Figure 1:**
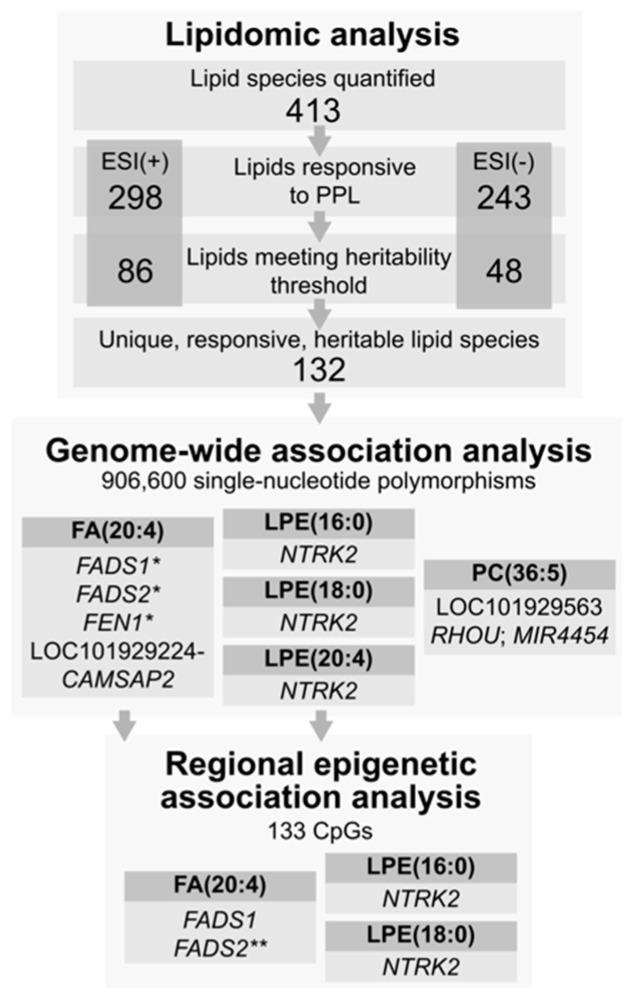
Summary of study design and findings. * replicated in an external cohort. ** evidence of partial mediation of SNP association by a CpG.

The GOLDN cohort was 50% male with an average age of 49 years (Table 1). The mean body mass index and cholesterol levels were within the normal range at fasting. Demographics and lipids were, on average, similar in the replication cohort. Among the ESI (+) mode, 298 lipids were responsive to the PPL, of which 86 were also heritable. On the ESI (−) mode, 243 lipids were PPL responsive and 48 of those were heritable. Among the 134 responsive and heritable lipids (see Supplemental Table S1), 132 were unique species across each mode (PE (36:4) and (38:4) being common). (See Figure 1.) Among the 132 unique lipid response traits, the three most heritable were PC (28:0), (30:1), and (30:1) (h^2^ ≥ 0.60). LPEs (22:6) and (16:0) as well as acylcarnitine (12:0) were among the most responsive to the PPL challenge (Supplemental Table S1).

After correction for multiple testing of 132 lipids (α = 5 × 10^−8^/132 = 4 × 10^−10^), no SNP was statistically significantly associated with any lipid. The top GWAS results for the PPL are presented in Table 2. Replication results from HAPI Heart are provided in the last three columns where data were available. The Manhattan and QQ plots for the lipids presented in Table 2 are shown in Supplemental Figures S1–S5. We found a marginal association in a known lipid locus around fatty acid desaturase 1 and 2 (*FADS1* and *FADS2*) on chromosome 11 for arachidonic acid (FA (20:4)). The four SNPs replicated after correction for multiple testing in consideration of that locus (*p* < 0.05/4 SNPs tested for replication in the *FADS1/2* locus). Two SNPs in *NTRK2* were also marginally associated with three LPE traits in GOLDN but were not available for replication in HAPI Heart. We then expanded the replication to all SNPs 5kb up and downstream of rs12552641 on chromosome 9 which were available in HAPI Heart. None of the SNPs replicated for LPE (16:0), (18:0), or (22:6) with *p* < 0.05. Those results are presented in Supplemental Table S2. Results from the sensitivity analyses in LMEKIN are presented in Supplemental Table S3.

We followed up the top GWAS findings (Table 2) with regional epigenetic analyses. Those results are presented in Supplemental Table S4, representing 133 CpGs, with *p*-values for association ranging from 5.2 × 10^−7^ to 0.98. In Table 3, we present the regional epigenetic results for the marginally associated (*p* < 0.01) CpGs from Supplemental Table S4, representing 2 CpGs in *NTRK2* for LPE (16:0) and LPE (18:0) and 14 in *FADS1/2* for arachidonic acid. The association of those 16 CpGs with the nearest GWAS SNP (rs12552641, rs174547, or rs174577) is also presented in Table 3. The two strongest CpG association findings were with arachidonic acid for cg27386326 (*p* = 5.2 × 10^−7^) and cg19610905 (*p* = 8.7 × 10^−6^). Upon SNP-CpG analysis, rs174577 was a strong meQTL for both CpGs (*p* = 1.0 × 10^−22^ for cg27386326 and 4.1 × 10^−93^ for cg19610905, respectively). Those two CpGs were examined for mediation with results presented in Table 4. We found that cg19610905 mediated ~24% (95% CI 7.2–41.2%) of the relationship between rs174577 and arachidonic acid (*p* = 0.005). Cg27386326 was not a mediator of the rs174577-arachidonic acid relationship. Finally, while rs174547 was a meQTL for cg07689907, that CpG did not mediate the rs174547-arachidonic acid association.

## 5. Discussion

Postprandial lipemia measures can give additional insight into dyslipidemia and cardiovascular risk related to chronic inflammation. Lipid levels vary due to the complex interplay of individual genes and diet but the mechanisms are difficult to understand in populations partly due to wide variations in diet. To better understand how genes may affect postprandial traits, we undertook a genetic study of lipidomic species that responded to a standardized high-fat meal in the familial GOLDN study. Among the lipidomic species, the majority changed in response to the meal, and a third of the responsive lipids were also heritable. We conducted GWAS on 132 heritable and responsive lipids and the strongest and replicated findings were in the well-characterized *FADS1/2* locus on chromosome 11. Regional methylation analysis also showed CpG associations at that locus. For one SNP (rs174577), we report partial mediation of the lipid association by a nearby CpG (cg19610905). Other novel findings included the biologically plausible *NTRK2* locus.

The *FADS1/2/3* gene cluster (including nearby *FEN1*) is a known lipid locus containing fatty acid desaturase 1 and 2 on chromosome 11 [50]. The function of *FADS3* is unknown, whereas *FADS1* and *FADS2* encode fatty acid desaturases facilitating conversion of dietary linoleic and α-linolenic acids into arachidonic and eicosapentaenoic acids, respectively. These enzymes have both inflammatory and anti-inflammatory properties and are considered therapeutic targets for cardiovascular disease, cancer, and inflammation [50,51,52]. Relevant to the GOLDN dietary intervention (~83% fat from heavy cream), lipid loading meals can invoke a transitory inflammatory response, in which arachidonic acid appears to play a key role [53,54]. Most arachidonic acid comes from dietary linoleic acid derived from oils and animal fats [53]. In the cytoplasm, arachidonic acid can be modified by 5-lipoxygenase into leukotrienes or by cyclooxygenases into prostaglandin H2 that subsequently serves as the substrate for enzymatic pathways leading to the production of prostaglandins and thromboxanes, potent mediators of inflammation [50]. Importantly, variation in *FADS1* and *FADS2* is known to contribute to inter-individual differences in arachidonic acid levels and other long chain polyunsaturated fatty acids (PUFAs) [55]. For instance, the SNP rs174547 is located in intron 9 of *FADS1* [56]. Several GWAS previously described rs174547 as being associated with fatty acids [57,58,59]. A Mendelian Randomization study using rs174547 as an instrument reported higher genetically predicted plasma phospholipid arachidonic acid concentrations were associated with increased risk of colorectal and lung cancer [60]. Functional follow-up of that SNP shows it is associated with decreased *FADS1* expression in the human liver [61]. Several studies have focused on this variant and its association with omega 6 PUFA levels [62,63,64]. Other human studies have also noted allele-specific methylation around this locus in liver [65,66], and white blood cells [67] in smaller samples than GOLDN (*N* < 100). However, previous studies have not focused on the response to a standardized high-fat meal or how SNPs and CpGs may interplay to affect these responses. When examining CpGs in the *FADS1/2/3* region, cg07689907 was associated with arachidonic acid (*p* = 9 × 10^−4^), exceeding the Bonferroni correction for multiple testing for the 46 CpGs in that gene region (*p* < 0.0001). Rs174547 was a meQTL for cg07689907 with *p* = 6.9 × 10^−29^. Further analysis showed cg07689907 did not mediate the relationship between rs147547 and arachidonic acid. However, we report another arachidonic acid-associated CpG (cg19610905, *p* = 8.7 × 10^−6^) partially mediated the relationship between nearby rs174577 and the arachidonic acid lipid response. Since methylation is a driver of gene expression, it is possible that the functional effects of these polymorphisms may be through epigenetic processes. Another study set in an Asian population demonstrated rs174570 in *FADS1* was associated with gene expression through methylation in adipose tissue [68]. These findings provide novel evidence that both SNPs and CpGs in this region exert a joint and independent influence on these dietary response traits adding to their complexity.

Our other top findings were intronic to neurotrophic receptor tyrosine kinase 2 (*NTRK2*) for LPE (16:0), (18:0), and (22:6). LPEs are considered to be a minor lipid in milk and have been shown to respond to a dairy meal [69]. The neurotrophins are a family of growth factors known to be involved in the development, maintenance, and function of peripheral and central neurons and are hypothesized to play an important role in mediating neuronal plasticity in the hypothalamus. The neurotrophin receptor, TrkB, and its natural ligand, brain-derived neurotrophic factor (BDNF), have been implicated in the regulation of food intake and body weight [70]. Homozygous null mutations in *NTRK2*, the gene encoding TrkB, are lethal in rodents [71]. These variants were not available in the HAPI Heart data; thus, they were not replicated. Though we saw some marginal association of CpGs nearby these variants with the traits, these SNPs were not meQTLs and we did not test for mediation.

The main strength of this study included available data on responsive lipidomic phenotypes from a standardized meal in over 1200 individuals for an unprecedented study of gene-diet interaction. Unfortunately, some low-frequency (<1%) population variants in *NTRK2* present in GOLDN were not present in HAPI Heart, demonstrating the challenges of replication of rarer variants in small sample sizes. HAPI Heart did not have methylation data on the samples with lipidomics to replicate regional CpG findings or the mediation analysis in the region of the *FADS1/2* locus.

In conclusion, genetic factors are known to contribute to variation in response to dietary exposures. Especially high-fat meals may lead to temporary inflammatory states. Notably, cumulative exposure to chronic inflammation leads to cardiovascular risk and as well as other chronic diseases (e.g., cancer). Overall, the genetic mechanisms that dictate these responses are incompletely understood. In the current study, we report new information on the *FADS1/2* locus showing both SNPs and CpGs contribute to its effects on diet response. Though genetic variation is largely stable, CpGs may be modified by external factors over time. Future studies should investigate CpG loci in the region over the lifespan to determine if they contribute to changes in the metabolism of high-fat meals with aging, which could influence cardiovascular risk.

## Figures and Tables

**Table 1:** Demographic and clinical characteristics of the HAPI Heart cohort and the GOLDN cohort.

	GOLDN	HAPI Heart
*N*	668	639
Sex, Male	50%	56%
Age (years)	49 (16)	43 (13)
BMI (kg/m^2^)	28 (5)	26 (4)
Cholesterol (mg/dL)	193 (40)	207 (46)
Fasting LDL (mg/dL)	124 (32)	138 (42)
Fasting HDL (mg/dL)	47 (13)	55 (14)
Fasting Triglycerides (mg/dL)	141 (100)	67 (40)
6 h Triglycerides (mg/dL)	241 (186)	173 (127)
Data recorded as Mean (SD) or %		

BMI: body mass index; LDL: low-density lipoprotein; HDL: high-density lipoprotein.

**Table 2:** Top GWAS findings from the GOLDN lipidomics discovery effort (PPL intervention) and replication in the HAPI Heart Study.

Lipid	Variant Information				GWAS Discovery Results in GOLDN			Replication Results in HAPI Heart		
	CHR/SNP	BP Build38	Gene	Functional Annotation	EA/RA/EAF	SNP Beta (SE)	*p*-Value *	EA/RA/EAF	SNP Beta (SE)	*p*-Value
FA (20:4)	11/rs174547	61,803,311	*FADS1*	intronic	C/T/0.29	−0.26 (0.05)	1.68 × 10^−7^	C/T/0.23	−1.20 × 10^−1^ (0.04)	1.52 × 10^−3^
FA (20:4)	11/rs174577	61,837,342	*FADS2*	intronic	A/C/0.28	−0.26 (0.05)	1.39 × 10^−7^	A/C/0.25	−1.08 × 10-^1^ (0.04)	3.27 × 10^−3^
FA (20:4)	11/rs174583	61,842,278	*FADS2*	intronic	T/C/0.28	−0.24 (0.04)	7.03 × 10^−7^	T/C/0.25	−1.08 × 10^−1^ (0.04)	3.27 × 10^−3^
FA (20:4)	11/rs4246215	61,796,827	*FEN1*	UTR3	T/G/0.29	−0.25 (0.04)	1.22 × 10^−7^	T/G/0.26	−8.77 × 10^−2^ (0.04)	1.84 × 10^−2^
FA (20:4)	1/rs12042035	200,710,954	*LOC101929224-CAMSAP2*	intergenic	G/T/0.01	0.76 (0.12)	6.31 × 10^−9^	-	-	-
LPE (16:0)	9/rs12552641	84,838,137	*NTRK2*	intronic	G/C/0.01	1.83 (0.31)	4.38 × 10^−8^	-	-	-
LPE (16:0)	9/rs12555204	84,838,239	*NTRK2*	intronic	G/A/0.01	1.83 (0.31)	4.43 × 10^−8^	-	-	-
LPE (18:0)	9/rs12552641	84,838,137	*NTRK2*	intronic	G/C/0.01	1.67 (0.31)	3.78 × 10^−7^	-	-	-
LPE (18:0)	9/rs12555204	84,838,239	*NTRK2*	intronic	G/A/0.01	1.67 (0.31)	3.88 × 10^−7^	-	-	-
LPE (22:6)	9/rs12555204	84,838,239	*NTRK2*	intronic	G/A/0.01	1.68 (0.32)	9.49 × 10^−7^	-	-	-
LPE (22:6)	9/rs12552641	84,838,137	*NTRK2*	intronic	G/C/0.01	1.68 (0.32)	9.50 × 10^−7^	-	-	-
PC (36:5) A	9/rs10965980	23,625,667	*LOC101929563*	ncRNA_intronic	T/C/0.01	1.53 (0.23)	2.60 × 10^−9^	T/C/0.02	1.21 × 10^−1^ (0.18)	5.14 × 10^−1^
PC (36:5) A	9/rs10965998	23,634,930	*LOC101929563*	ncRNA_intronic	G/A/0.01	1.53 (0.23)	2.90 × 10^−9^	G/A/0.02	1.21 × 10^−1^ (0.18)	5.14 × 10^−1^
PC (36:5) A	9/rs10965994	23,634,530	*LOC101929563*	ncRNA_intronic	A/T/0.01	1.40 (0.25)	8.09 × 10^−7^	A/T/0.02	1.21 × 10^−1^ (0.18)	5.14 × 10^−1^
PC (36:5) A	9/rs17836336	23,619,256	*LOC101929563*	ncRNA_intronic	G/C/0.01	1.55 (0.26)	1.34 × 10^−7^	-	-	-
PC (36:5) A	1/rs431675	229,169,403	*RHOU;MIR4454*	intergenic	C/T/0.38	0.25 (0.04)	3.51 × 10^−9^	C/T/0.36	−3.05 × 10^−2^ (0.18)	5.62 × 10^−1^

Abbreviations: PPL-postprandial lipemia; BP-base pair; CHR-chromosome; FA (20:4)-arachidonic acid; GWAS-genome-wide association study; LPE-lysophosphatidylethanolamine; PC-phosphatidylcholine; SE-standard error, SNP-single-nucleotide polymorphism; EA-Effect Allele; RA-Reference Allele; EAF-Effect Allele frequency * inflation corrected.

**Table 3:** Regional CpG-lipid associations and meQTLs in GOLDN.

Lipid	Gene	CpG	CHR:BP (hg38)	CpG Beta (SE)	CpG *p*-Value	SNP	Distance from CpG	meQTL Beta (se)	Z	meQTL *p*-Value
LPE (16:0)	*NTRK2*	cg13504245	9:84667694	−2.41 (0.90)	7.44 × 10^−3^	rs12552641	+170,443	−1.50 × 10^−2^ (1.28 × 10^−2^)	−1.17	2.42 × 10^−1^
LPE (18:0)	*NTRK2*	cg14447193	9:84818948	−2.45 (0.85)	5.47 × 10^−3^	rs12552641	+19,189	−3.32 × 10^−2^ (1.35 × 10^−2^)	−2.45	1.46 × 10^−2^
FA (20:4)	*FADS1*	cg25448062	11:61812645	−3.46 (1.32)	8.83 × 10^−3^	rs174547	+9334	−6.40 × 10^−3^ (6.59 × 10^−4^)	−9.70	6.79 × 10^−21^
FA (20:4)	*FADS1*	cg07689907	11:61815101	1.50 (0.45)	9.05 × 10^−4^	rs174547	−11,790	−4.09 × 10-^2^(3.49 × 10^−3^)	−11.71	6.87 × 10-^29^
FA (20:4)	*FADS1*	cg14725641	11:61815290	7.20 (2.63)	6.22 × 10^−3^	rs174547	−11,979	−4.58 × 10^−3^ (5.44 × 10^−4^)	−8.43	2.22 × 10^−16^
FA (20:4)	*FADS1*	cg25326896	11:61815312	8.59 (2.81)	2.26 × 10^−3^	rs174547	−12,001	−3.42 × 10^−3^ (5.48 × 10^−4^)	-6.24	7.97 × 10^−10^
FA (20:4)	*FADS1*	cg12517394	11:61815322	5.60 (2.14)	9.07 × 10^−3^	rs174547	−12,011	−4.21 × 10^−3^ (6.51 × 10^−4^)	−6.47	1.97 × 10^−10^
FA (20:4)	*FADS1*	cg15598662	11:61815417	7.19 (2.13)	7.28 × 10^−4^	rs174547	−12,106	3.42 × 10^−3^ (1.12 × 10-3)	3.04	2.44 × 10^−3^
FA (20:4)	*FADS1*	cg23992449	11:61816483	14.82 (5.08)	3.57 × 10^−3^	rs174547	−13,172	−7.17 × 10^−4^ (2.60 × 10^−4^)	−2.75	6.06 × 10^−3^
FA (20:4)	*FADS1*	cg27173322	11:61816639	17.47 (4.99)	4.68 × 10^−4^	rs174547	−13,328	−6.15 × 10^−4^ (2.72 × 10^−4^)	−2.26	2.42 × 10^−2^
FA (20:4)	*FADS2*	cg27386326	11:61820507	−2.03 (0.41)	5.18 × 10^−7^	rs174577	+16,835	4.57 × 10^−2^ (4.49 × 10^−3^)	10.18	1.01 × 10^−22^
FA (20:4)	*FADS2*	cg06781209	11:61827524	1.16 (0.41)	4.69 × 10^−3^	rs174577	+9818	−2.92 × 10^−2^ (4.44 × 10^−3^)	−6.58	9.61 × 10^−11^
FA (20:4)	*FADS2*	cg16576620	11:61828596	9.80 (3.45)	4.52 × 10^−3^	rs174577	+8746	−9.60 × 10^−4^ (4.01 × 10^−4^)	−2.40	1.69 × 10^−2^
FA (20:4)	*FADS2*	cg19610905	11:61828860	7.06 (1.59)	8.68 × 10^−6^	rs174577	+8482	−1.56 × 10^−2^ (6.45 × 10^−4^)	−24.20	4.07 × 10^−93^
FA (20:4)	*FADS2*	cg00603274	11:61829153	1.68 (0.55)	2.41 × 10^−3^	rs174577	+8189	−2.15 × 10^−2^ (2.91 × 10^−3^)	−7.38	4.87 × 10^−13^
FA (20:4)	*FADS2*	cg14911132	11:61829282	2.86 (1.02)	4.97 × 10^−3^	rs174577	+8060	−7.46 × 10^−3^ (1.48 × 10^−3^)	−5.05	5.86 × 10^−7^

Abbreviations: BP-base pair; CHR- chromosome; CpG-cytosine-phosphate-guanine site; FA (20:4)-arachidonic acid; LPE-lyso phosphatidylethanolamine; meQTL-methylation quantitative trait loc;i SE-standard error; SNP-single-nucleotide polymorphism.

**Table 4:** Mediation Analysis.

Outcome	Treatment	Mediator	Effect	Estimate	SE	95% CI		Z	*p*-Value
						Lower	Upper		
FA (20:4) (6hr diff)	rs174577	cg27386326	Total Effect	−0.24	0.04	−0.32	−0.15	−5.39	<0.0001
			Natural Direct Effect (Treatment)	−0.18	0.06	−0.29	−0.06	−2.98	0.0029
			Natural Indirect Effect (Mediator)	−0.06	0.04	−0.14	0.02	−1.57	0.1173
			Percentage Mediated	25.95	17.24	−7.84	59.74	1.51	0.1322
		cg19610905	Total Effect	−0.24	0.04	−0.32	−0.15	−5.39	<0.0001
			Natural Direct Effect (Treatment)	−0.18	0.05	−0.27	−0.09	−3.83	0.0001
			Natural Indirect Effect (Mediator)	−0.06	0.02	−0.09	−0.02	−3.18	0.0015
			Percentage Mediated	24.17	8.69	7.15	41.20	2.78	0.0054
	rs174547	cg07689907	Total Effect	−0.24	0.04	−0.32	−0.15	−5.36	<0.0001
			Natural Direct Effect (Treatment)	−0.22	0.05	−0.31	−0.12	−4.50	<0.0001
			Natural Indirect Effect (Mediator)	−0.02	0.02	−0.06	0.02	−0.96	0.3394
			Percentage Mediated	7.99	8.49	−8.65	24.62	0.94	0.3468

Abbreviations: FA (20:4)-arachidonic acid; SE-standard error; CI-confidence interval.

## Data Availability

Data available on request due to restrictions.

## Supplementary Materials

The following are available online at https://www.mdpi.com/article/10.3390/nu13114000/s1, Figure S1. Arachidonic acid, FA (20:4) Manhattan and QQ plot. Figure S2. LPE (16:0) Manhattan and QQ plot; Figure S3. LPE (18:0) Manhattan and QQ plot; Figure S4. LPE (22:6) Manhattan and QQ plot; Figure S5. PC (36:5)A Manhattan and QQ plot; Table S1. GOLDN responsive and heritable lipid species Table S2. Expanded replication on chromosome 9 in HAPI Heart; Table S3. Results of the GOLDN sensitivity analysis; Table S4. Regional epigenetic analysis of GWAS findings from Table 2.
