# Supplementary material for: Genomics of Postprandial Lipidomics in the Genetics of Lipid-Lowering Drugs and Diet Network Study
Source: Nutrients. 2021 Nov 10;13(11):4000. doi: 10.3390/nu13114000 (PMC8617762; doi:10.3390/nu13114000)

Figure S1. Arachidonic acid, FA (20:4) Manhattan and QQ plot.

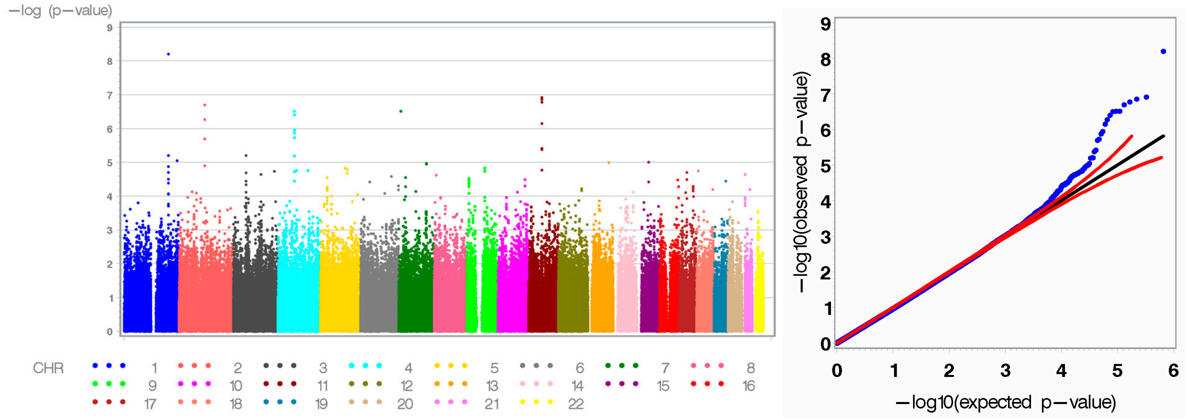


Figure S2. LPE (16:0) Manhattan and QQ plot

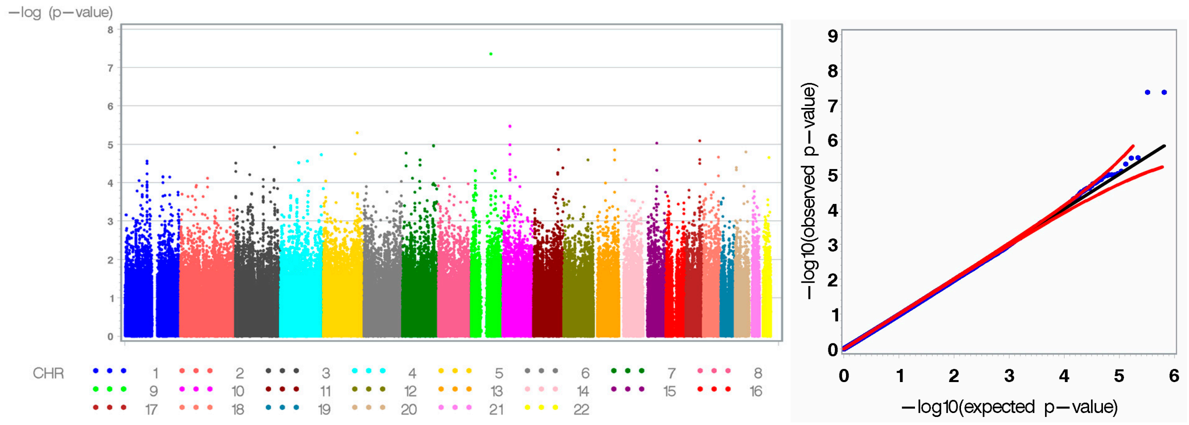

Figure S3.  LPE (18:0) Manhattan and QQ plot

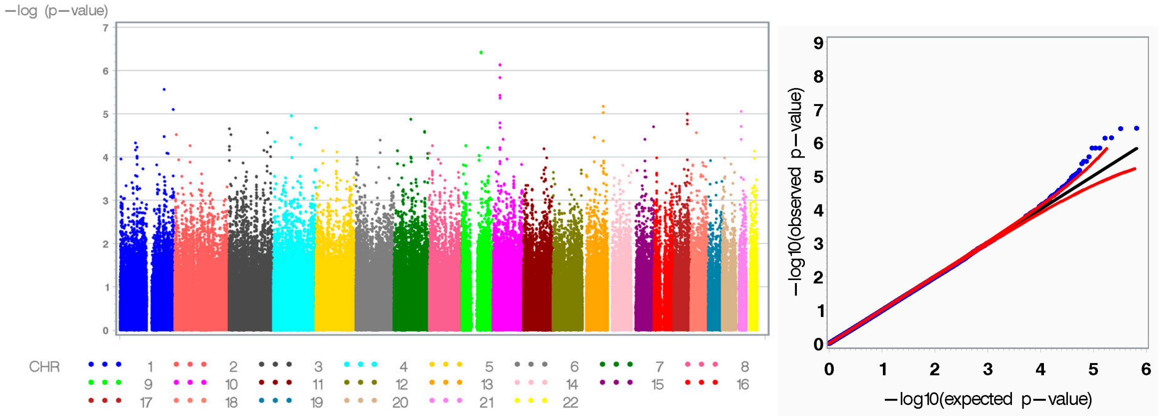


Figure S4.  LPE (22:6) Manhattan and QQ plot

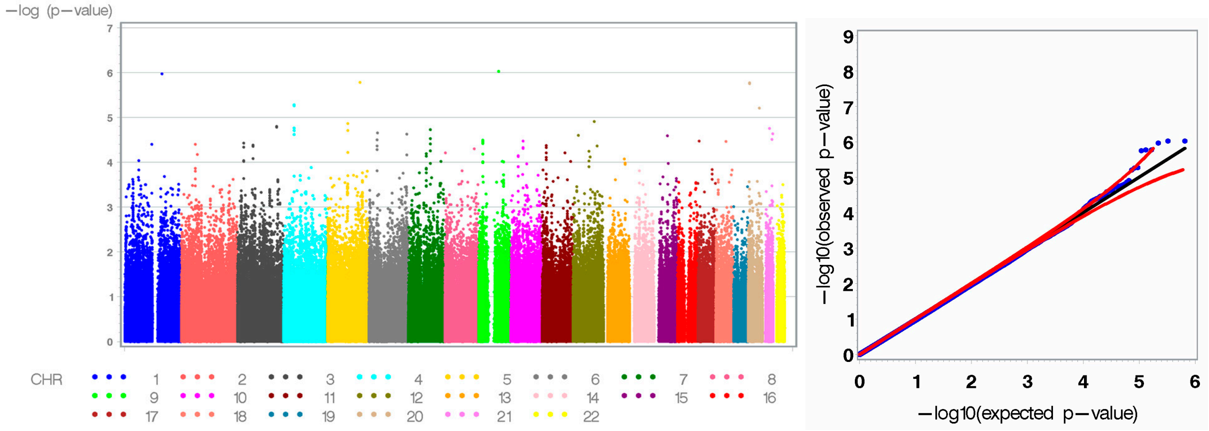

Figure S5. PC (36:5)A Manhattan and QQ plot

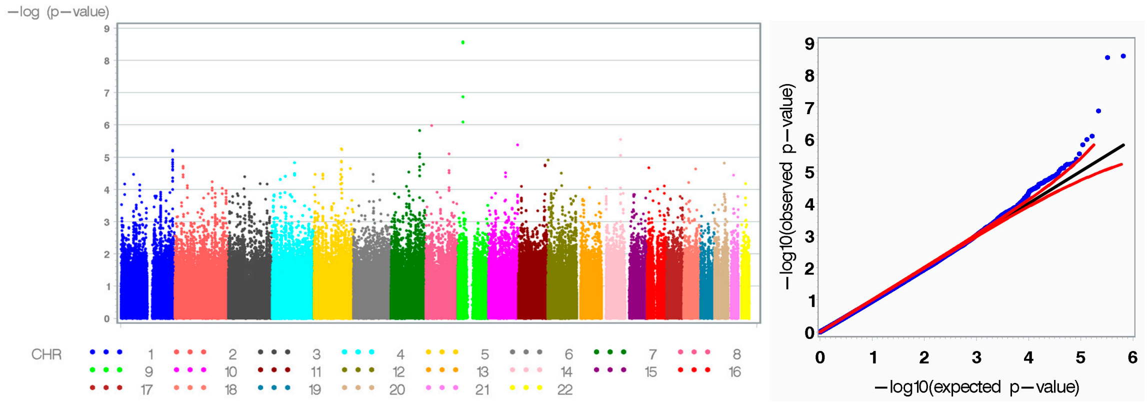

Supplement: Supplementary file 1 [file nutrients-13-04000-s001.zip › nutrients-1444395-supplementary/Irvin Arnett et al Supplement/Irvin Arnett et al GOLDN PPL lipidomics SupplementalFigures1_5.pdf]
